# Isorhamnetin protects porcine oocytes from zearalenone-induced reproductive toxicity through the PI3K/Akt signaling pathway

**DOI:** 10.1186/s40104-022-00809-w

**Published:** 2023-02-03

**Authors:** Xiaoya Li, Jiaxin Duan, Shiyou Wang, Jianyong Cheng, Huali Chen, Zelin Zhang, Li Yang, Rongmao Hua, Qingwang Li

**Affiliations:** 1 College of Animal Science and Technology, Northwest A&F University, Yangling, 712100 People’s Republic of China; 2 College of Animal Science and Technology, Shanxi Agricultural University, Taigu, 030031 People’s Republic of China; 3 School of Life Science and Engineering, Southwest University of Science and Technology, Mianyang, 621000 People’s Republic of China; 4 College of Pharmacy, Shenzhen Technology University, Shenzhen, 518118 People’s Republic of China

**Keywords:** Apoptosis, Isorhamnetin, Oocyte, Oxidative stress, Porcine, Zearalenone

## Abstract

**Background:**

Zearalenone (ZEA) widely exists in moldy grains, which seriously destroys the fertility of females. Isorhamnetin, a natural flavonoid, has extensive of pharmacological activities. However, the beneficial effect and the underlying molecular mechanism of isorhamnetin involvement in ZEA-induced porcine oocyte damage have not been investigated.

**Methods:**

Oocytes were treated with different concentrations of ZEA (3, 5, 8 and 10 μmol/L) and isorhamnetin (5, 10, 20 and 30 μmol*/*L) for 44 h at 39 ℃. ZEA (5 μmol/L) and isorhamnetin (10 μmol/L) were selected for subsequent studies. Polar body exclusion rate, apoptosis rate and apoptosis related proteins, ROS levels and SOD2 protein, mitochondrial membrane potential and distribution, endoplasmic reticulum distribution and proteins expression, and PI3K, Akt and p-Akt proteins expression of oocytes were detected. In addition, the effect of PI3K antagonist (LY294002) on oocyte nuclear maturation and apoptosis were used to determine the involvement of PI3K/Akt signaling pathway.

**Results:**

Our findings showed that ZEA exposure damaged oocytes and isorhamnetin therapy restored the developmental capability of porcine oocytes. Isorhamnetin promoted polar body extrusion rate to rescue ZEA-induced meiotic arrest in porcine oocytes. Isorhamnetin alleviated ZEA-induced oxidative stress by stimulating SOD2 protein expression and inhibiting ROS production. Moreover, isorhamnetin enhanced normal mitochondrial distribution and mitochondrial membrane potential to prevent mitochondrial dysfunction induced by ZEA. Changing the expression of endoplasmic reticulum stress-related marker proteins (CHOP, GRP78) and the distribution rate of normal endoplasmic reticulum showed that isorhamnetin relieved ZEA-caused endoplasmic reticulum stress. Mechanistically, isorhamnetin decreased Bax/Bcl-2 protein expression and inhibited ZEA-induced apoptosis through PI3K/Akt signaling pathway.

**Conclusions:**

Collectively, these results suggest that isorhamnetin protects oocytes from ZEA-caused damage through PI3K/Akt signaling pathway, which enhances meiotic maturation and mitochondrial function, and inhibits early apoptosis, oxidative stress and endoplasmic reticulum stress in porcine oocytes. Our study provides a new strategy for solving the reproductive toxicity induced by ZEA and treating woman infertility.

**Graphical Abstract:**

A possible mechanism by which isorhamnetin protected porcine oocytes from ZEA-induced damage. Isorhamnetin inhibited meiosis arrest and apoptosis of porcine oocytes induced by ZEA through the PI3K/Akt signaling pathway. Moreover, isorhamnetin repaired ZEA-induced oocyte damage by alleviating oxidative stress, mitochondrial dysfunction and ER stress.

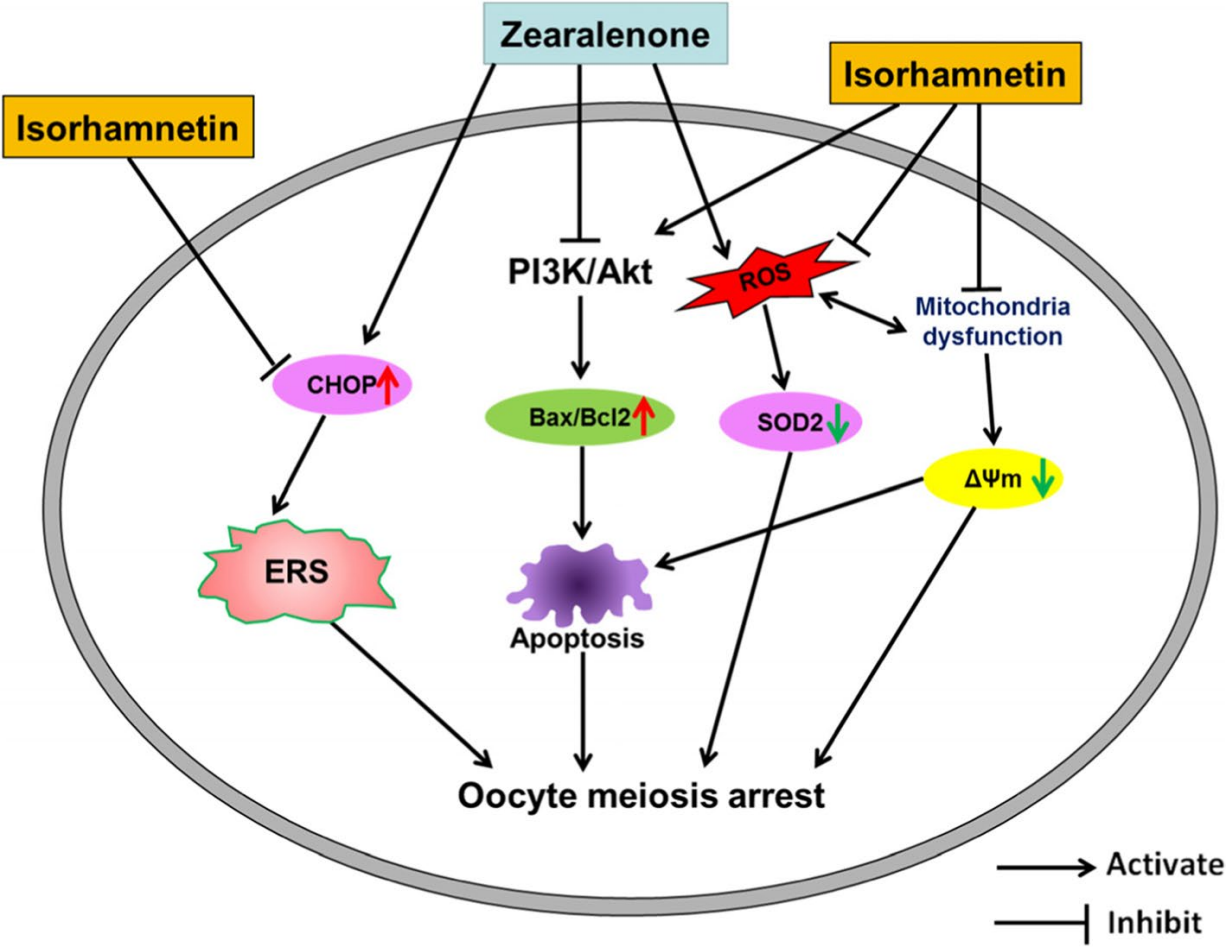

**Supplementary Information:**

The online version contains supplementary material available at 10.1186/s40104-022-00809-w.

## Introduction

Mammalian oocytes provide most of the maternal genetic materials, cytoplasmic components, organelles and membranes for embryonic development [1]. Oocytes must undergo chromosome division and cytoplasm division to reach maturation [2]. High quality oocytes are essential for successful fertilization, early embryo survival and healthy fetal development [3]. However, many defects can damage oocyte maturation, including oxidative stress, apoptosis, endoplasmic reticulum (ER) stress and mitochondrial dysfunction [4–6]. Thus, the quality of oocytes is the major determinant of female reproductive potential.

Zearalenone (ZEA), a mycotoxin like estrogen, is widely distributed in contaminated foods such as corn, oats and millet [7, 8]. ZEA induces endocrine disorders and leads to a variety of reproductive diseases by binding to estrogen receptors [9]. Studies have shown that ZEA impaired the development of human and animal sperm and oocytes [10]. ZEA disrupted normal pregnancy in female mice by interfering with fertilization rate and embryo development [11]. Pigs are more sensitive to ZEA-induced reproductive damage than other domestic animals [12]. Malekinejad et al. reported that ZEA inhibited the meiosis of porcine oocytes by inducing spindles malformation [13]. ZEA interfered with the initial chromatin state and destroyed meiotic competence of gilt oocytes [14]. Moreover, ZEA induced abnormal mitochondrial distribution, mitochondrial membrane potential damage and ER stress to impair meiotic maturation of porcine oocytes [15]. ZEA also inhibited the polar body extrusion rate of porcine oocytes by stimulating ROS production [16]. Given ZEA's heat stability (up to 160 °C), it was impossible to completely eradicate in the food chain, which could be harmful to humans [12]. At present, there is no effective antidote for ZEA. Thus, it is necessary to explore potential compounds that can effectively protect ZEA-induced oocyte damage.

Isorhamnetin, a flavonoid compound, is present in man’s daily diet, such as pears [17], onion [18] and peanut [19]. Isorhamnetin is known for its pharmacological activities such as antioxidant [20], antitumor [21], anti-inflammatory [22], antiviral [23], antiosteoporosis [24] and antihyperuricemia [25], etc. Natural flavonoids have attracted extensive attention because of their antioxidant activity to promote oocyte development. Growing evidences have revealed that quercetin [26] and kaempferol [27] promoted oocyte maturation by inhibiting oxidative stress. Our previous results suggested that isorhamnetin promoted estrogen secretion and proliferation in porcine ovarian granulosa cells [28]. In addition, previous studies in our laboratory showed that isorhamnetin could promote the development of porcine oocytes. The above results hint that isorhamnetin may promote the development of oocytes. However, it is unclear whether isorhamnetin can protect porcine oocytes from ZEA-caused damage.

PI3K/Akt signaling pathway has the functions of regulating oocyte maturation [29]. Studies have shown that iloprost enhanced porcine embryo development through PI3K/Akt signaling pathway [30]. Our previous studies also showed isorhamnetin promoted the proliferation of porcine ovarian granulosa cells by activating PI3K/Akt signaling pathway [28]. Whole-transcriptome sequencing technology indicated that ZEA-induced porcine granulosa cells cycle arrest through the PI3K/Akt signaling pathway [31]. However, the role of PI3K/Akt and isorhamnetin in ZEA-caused damage have not been studied.

In this study, we explored whether isorhamnetin could exert a protective effect on ZEA-induced damage in porcine oocytes. Moreover, the signaling pathway of isorhamnetin involved in this cytoprotective effect was studied. We found that isorhamnetin therapy suppressed ZEA-induced damage by promoting meiotic maturation and mitochondrial function, and inhibiting early apoptosis, oxidative stress and ER stress in porcine oocytes. Our results show that isorhamnetin acts as a natural and non-toxic additive to protect ZEA-induced damage in porcine oocytes.

## Materials and methods

### Chemical

Isorhamnetin (Cat#:N1358, ApexBio, Houston, Texas, USA) was primely dissolved in dimethyl sulfoxide (DMSO) and stored at − 20 °C. The concentration of isorhamnetin stock solution was 100 mmol/L. Before use, the final concentration of isorhamnetin was diluted with culture medium. PI3K antagonist (LY294002, Cat#:S1737) was obtained from Beyotime (Shanghai, China). Antibodies information: GAPDH (Cat#:60004–1-1g) and goat anti-rabbit (Cat#:31460) /mouse (Cat#:31430) IgG (H + L)-HRP were purchased from Proteintech (Wuhan, Hubei province, China); Bcl-2 (Cat#:01556), Cleaved caspase-3 (Cat#:02117), Bax (Cat#:01637), SOD2 (Cat#:02506), p-Akt (Cat#:P001a), Akt (Cat#:0003b), PI3K (Cat#:02894), GRP78 (Cat#:03157) and CHOP (Cat#:00880) were purchased from WanleiBio (Shenyang, Liaoning province, China).

### Culture of porcine cumulus oocyte complexes (COCs)

Ovaries were obtained from a local abattoir (Yangling, Shaanxi province, China) and stored in 37 °C phosphate-buffered saline (PBS) containing 1% antibiotic. Follicular fluid was harvested from healthy antral follicles using a 10-mL disposable syringe (26-gauge needle). More than three layers of COCs were collected by stereomicroscopy. COCs were washed two or three times in TCM199 (Gibco, Grand Island, State of New York, USA) containing 10 mmol/L 4-(2-hydroxy-ethyl)-1-piperazineethanesulfonic acid and 5% fetal bovine serum (FBS, Serapro, Meschede, North Rhine-Westphalia, Germany). Every 50–60 COCs was transferred into 500 μL TCM199 medium with 10% FBS, 10 IU/mL human chorionic gonadotropin (Ningbo Second Hormone, Ningbo, Zhejiang province, China), 1% antibiotics (penicillin−streptomycin solution), 0.57 mmol/L *L*-cysteine, 10 IU/mL pregnant mare serumgona dotropin (Ningbo Second Hormone), 0.91 mmol/L sodium pyruvate, 10% porcine follicle fluid, 10 IU/mL follicle stimulating hormone (Ningbo Second Hormone), 1 mg/mL poly(vinyl alcohol), 3.05 mmol/L glucose and 10 ng/mL epidermal growth factor for 44 h at 39 ℃ in 5% CO_2_. Other chemicals and reagents not mentioned were purchased from sigma Aldrich (St. Louis, Missouri, USA). According to the nuclear maturation rate of oocytes and previous reports [15, 29], ZEA (3, 5, 8, and 10 μmol/L) with or without isorhamnetin (5, 10, 20, and 30 μmol/L), and PI3K antagonist (LY294002, 20 μmol/L) were added in medium, respectively.

### In vitro maturation (IVM) of oocytes

After maturation (44 h), cumulus cells around oocytes were removed with 1 mg/mL hyaluronidase. Nextly, oocytes were cultured with Hoechst 33342 (Beyotime) for 25 min. Stained oocytes were washed three times (PBS) and examined with inverted fluorescence microscope (Olympus, Tokyo, Japan), oocytes with two chromatin bright spots were identified as mature oocytes. Each treatment group had 20–30 oocytes for maturation analysis. Data were obtained from at least three independent replicate experiments.

### Detection of early apoptosis by annexin-V staining

The denuded oocytes were incubated with 60 μL binding buffer containing 2.5 μL of Annexin-V-FITC (Beyotime) for 25 min. After washing three times (PBS), the apoptosis rate of positive oocytes was detected by inverted fluorescence microscope (Olympus). There were 50–60 oocytes for apoptosis detection in each treatment group. Data were obtained from at least three independent replicate experiments.

### Measurement of reactive oxygen species(ROS)

Oocytes were loaded with 2 μmol/L (ROS Assay Kit, WanleiBio) probe for 30 min. After washing, the fluorescence signal of oocytes was collected by fluorescence microscope (Olympus). The fluorescence intensity of the ROS signals was calculated with the ImageJ. Each treatment group had 50–60 oocytes for ROS fluorescence detection. Data were obtained from at least three independent replicate experiments.

### Mitochondrial membrane potential assay

Denuded oocytes were stained with 10 µmol/L JC-1 solution (Solarbio, Beijing, China) for 35 min. Then, the oocytes were washed and examined immediately under inverted fluorescence microscopy (Olympus). Microphotographs were calculated by ImageJ Software. Mitochondrial membrane potential was the ratio of red to green fluorescence intensity. 20–30 oocytes were tested for mitochondrial membrane potential. Data were obtained from at least three independent replicate experiments.

### Mitochondrial distribution evaluation

Oocytes were loaded with Mito-Tracker Red probe (200 mmol/L, Beyotime) for 45 min. After washing, oocytes were mounted on glass slides and observed by fluorescence microscopy (Olympus). Normal mitochondria were evenly distributed, while accumulative distribution, peripheral distribution and semi-peripheral distribution were considered to be abnormal mitochondrial distribution. 20–30 oocytes were tested for mitochondrial distribution. Data were obtained from at least four independent replicate experiments.

### Staining of ER in oocytes

Oocytes were loaded with ER-Tracker Blue probe (1 μmol/L, Solarbio) for 35 min. Oocytes were captured under fluorescence microscope (Olympus) after washing three times. Normal/abnormal endoplasmic reticulum was determined by the same method as mitochondria (2.8). 20–30 oocytes were tested for ER distribution. Data were obtained from at least four independent replicate experiments.

### Western blotting

100–150 oocytes were lysed in 16 μL lysis buffer (Beyotime) containing protease inhibitor. 5 × loading buffer (Beyotime) was mixed with each lysed sample and subsequently boiled at 100 ℃ for 8 min. Total lysate was size fractionated with sodium dodecyl sulfate–polyacrylamide gels and transferred to nitrocellulose membrane. Membrane was blocked in 5% skimmed milk powder for 1.5 h and incubated at 4 °C overnight with primary antibodies. Washing thrice (TBST), membrane was incubated with secondary antibody for 2 h. Membrane was washed and covered with 1:1 mix of chemiluminescence (Millipore, Boston, Massachusetts, USA). The band of protein was calculated by ImageJ software. Data were obtained from at least three independent replicate experiments.

### Statistical analysis

Statistical analyses was achieved with the Graphpad Prism (6.0) and SPSS (18.0) software. Data were showed as mean ± SEM and analyzed by one-way ANOVA followed by the Duncan’s test. The *P*-value less than 0.05 was considered to be significant. At least three biological replicates were used for each treatment.

## Results

### Isorhamnetin rescued nuclear maturation of ZEA exposed porcine oocytes

**Fig. 1:**
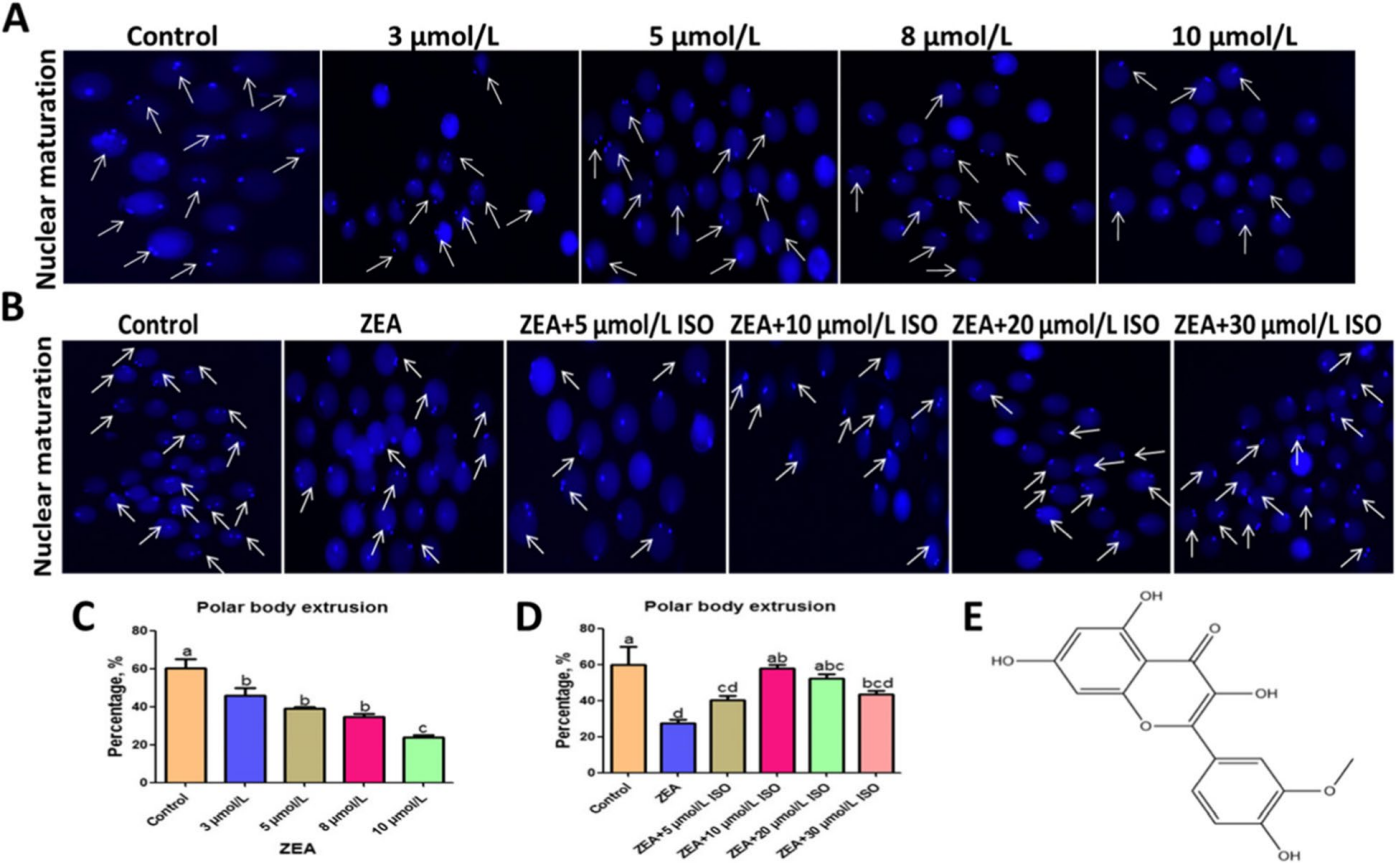
Isorhamnetin rescued nuclear maturation of ZEA exposed porcine oocytes. Oocytes were cultured with different concentrations ZEA (3, 5, 8, and 10 μmol/L) for 44 h with or without various concentrations of isorhamnetin (5, 10, 20 and 30 μmol/L). **A** Representative images of nuclear maturation treated with different concentrations of ZEA. White arrows indicate mature oocytes. **B** Effects of different concentrations of isorhamnetin on nuclear maturation of 5 μmol/L ZEA-damaged oocytes. **C** The polar extrusion rate of different ZEA treatment groups was recorded. **D** Percentage of polar extrusion treated with different concentrations of isorhamnetin and ZEA (5 μmol/L). **E** Structure of isorhamnetin. Various letters (*P* < 0.05) designate significant differences. Data are presented by average ± SEM

To investigate whether ZEA exposure affects oocyte maturation, porcine oocytes were treated with ZEA (3, 5, 8, and 10 μmol/L) for 44 h. Following ZEA treatment, oocyte maturation rate was decreased in a dose-dependent manner. As shown in Fig. 1A and C, the polar body extrusion rate of oocytes was significantly decreased from 60.28% ± 4.72% of control group, 45.83% ± 4.17% of 3 μmol/L group, 39.05% ± 0.95% of 5 μmol/L group, 34.85% ± 1.56% of 8 μmol/L group to 23.87% ± 1.14% of 10 μmol/L group (*P* < 0.05). Combined with previous studies [15] and the inhibitory effect of ZEA on oocyte maturation rate in this study, the 5 μmol/L of ZEA was selected as the concentration for subsequent studies.

To explore the beneficial effect of isorhamnetin on ZEA-caused damage, oocytes were co-cultured with ZEA (5 μmol/L) and isorhamnetin (5, 10, 20 and 30 μmol/L) during IVM. As indicated in Fig. 1B and D, the maturation rate of ZEA-exposed oocytes (35.61% ± 1.81%) was significantly lower than those of the control (60.14% ± 3.64%), ZEA + 10 μmol/L isorhamnetin (57.78% ± 2.23%) and ZEA + 20 μmol/L isorhamnetin (52.28% ± 2.28%) groups (*P* < 0.05). Particularly, the 10 μmol/L isorhamnetin was the most effective in repairing ZEA inhibition of oocyte maturation. However, there was no significant change in oocyte maturation rate between the ZEA group, ZEA + 5 μmol/L isorhamnetin group and ZEA + 30 μmol/L isorhamnetin group.

**Fig. 2:**
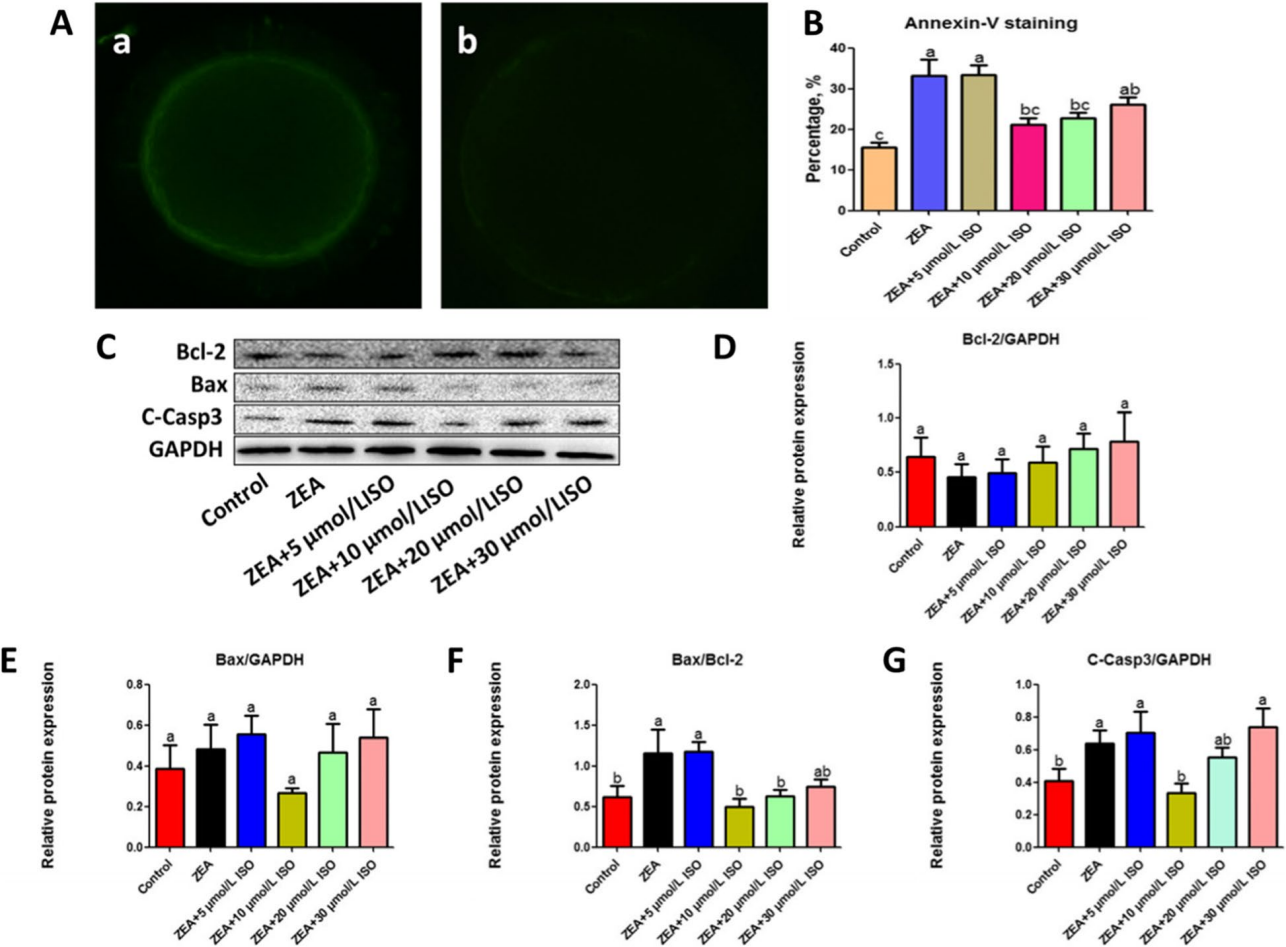
Isorhamnetin inhibited ZEA-caused apoptosis. Oocytes were cultured with ZEA (5 μmol/L) for 44 h with or without various concentrations of isorhamnetin (5, 10, 20 and 30 μmol/L). **A** Representative photomicrographs. (a) Early apoptotic oocyte. (b) Normal oocyte. **B** The ratios of early apoptosis was calculated at various groups. **C** Western blotting of Bcl-2, Bax and C-Casp3 were shown in different treatments. **D**–**G** The ratios of Bcl-2 to GAPDH, Bax to GAPDH, Bax to Bcl-2 and C-Caspase-3 to GAPDH expression were normalized, respectively. Various letters (*P* < 0.05) designate significant differences. Data are presented by average ± SEM

10 μmol/L and 20 μmol/L isorhamnetin also inhibited the apoptosis of oocytes exposed to 5 μmol/L ZEA (*P* < 0.01; Fig. 2A–B). Besides, the protein level of Bax/Bcl-2 and C-Casp3 were decreased in ZEA + 10 μmol/L isorhamnetin group compared with ZEA group (*P* < 0.05; Fig. 2C, F, and G). Altogether, these results showed that isorhamnetin improved the deficiency of ZEA-treated oocytes. Combined with our above results, 5 μmol/L ZEA and 10 μmol/L isorhamnetin were selected for the following experiments.

### Isorhamnetin attenuated ZEA-caused oxidative stress in porcine oocytes

**Fig. 3:**
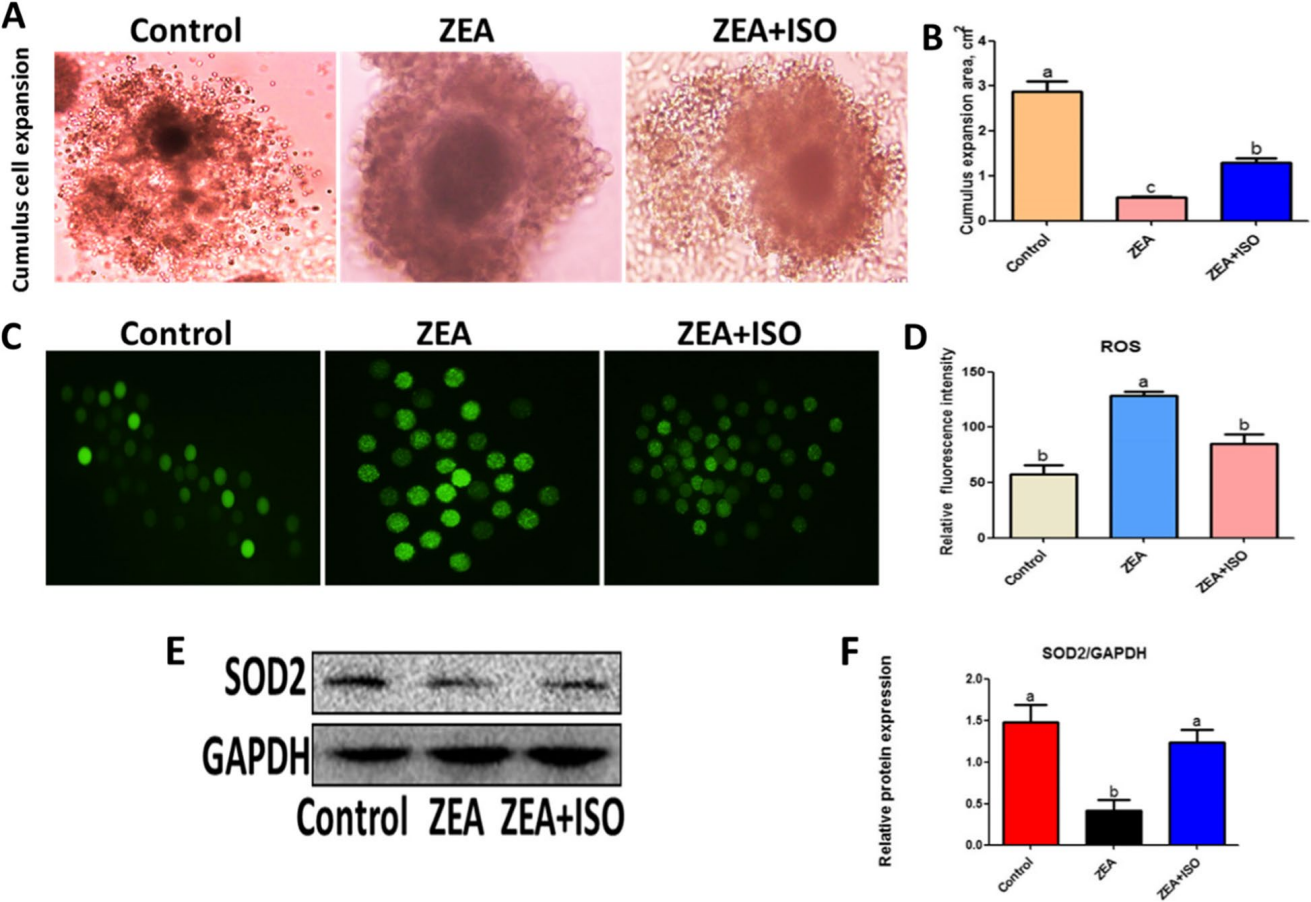
Isorhamnetin relieved oxidative stress caused by ZEA. Oocytes were treated with ZEA (5 μmol/L) for 44 h in the presence or absence of isorhamnetin (10 μmol/L). **A** Representative micrographs of cumulus cells expansion in Control, ZEA and ZEA + ISO. **B** The cumulus expansion area was calculated in Control, ZEA and ZEA + ISO. **C** Representative images of ROS fluorescence in Control, ZEA and ZEA + ISO. **D** The fluorescence intensity of ROS was measured. **E** Western blot of SOD2 protein in Control, ZEA and ZEA + ISO. **F** Ratios of SOD2 to GAPDH expression. Various letters (*P* < 0.05) designate significant differences. Data are presented by average ± SEM

Firstly, we observed that the expansion of cumulus cells in ZEA group was significantly lower than in the control group and ZEA + ISO group (Fig. 3A–B). To explore the role of isorhamnetin in alleviating ZEA-caused oxidative stress in porcine oocytes, the level of ROS and the relative expression of antioxidant-related protein was measured. After ZEA treatment (128.80% ± 3.61%) the level of ROS was significantly increased compared with the control group (57.55% ± 8.35%, *P* < 0.05; Fig. 3C–D), while the expression of antioxidant protein SOD2 was markedly decreased at ZEA group (*P* < 0.05; Fig. 3E–F). Fortunately, isorhamnetin treatment significantly counteracted ZEA-induced oxidative stress, indicating a significant reduction in ROS generation in porcine oocytes (*P* < 0.05; Fig. 3C–D). In addition, isorhamnetin effectively increased the decrease of SOD2 protein expression mediated by ZEA (*P* < 0.05; Fig. 3E–F), indicating that isorhamnetin prevents oxidative stress induced by ZEA.

### Isorhamnetin prevented ZEA-induced mitochondrial dysfunction in porcine oocytes

**Fig. 4:**
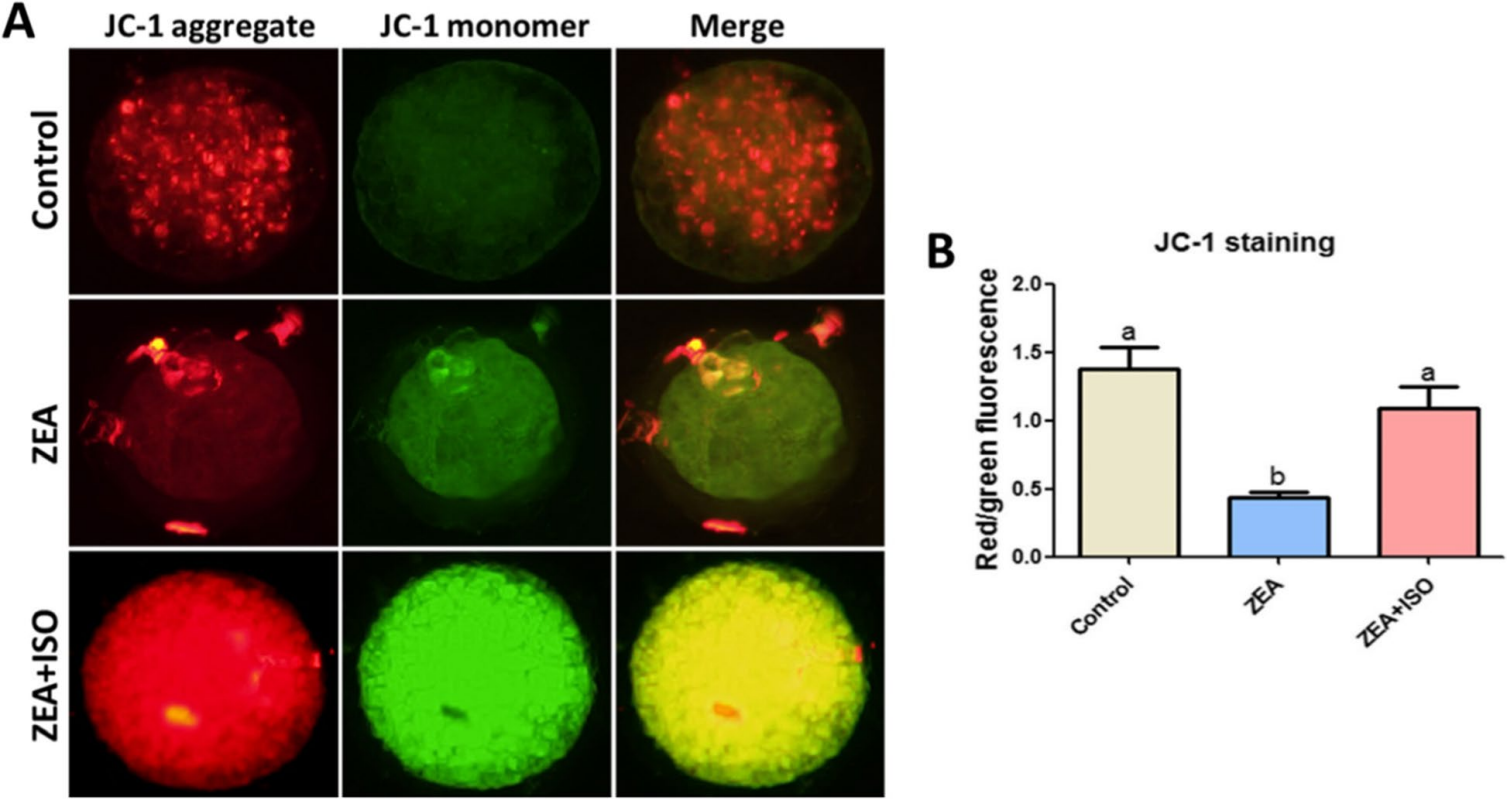
Isorhamnetin enhanced mitochondrial membrane potential in ZEA-exposed porcine oocytes. Oocytes were treated with ZEA (5 μmol/L) for 44 h in the presence or absence of isorhamnetin (10 μmol/L). **A** Representative pictures of mitochondrial membrane potential in Control, ZEA and ZEA + ISO. **B** The relative mitochondrial membrane potential is expressed as the ratio of red to green intensity. Various letters (*P* < 0.05) designate significant differences. Data are presented by average ± SEM

**Fig. 5:**
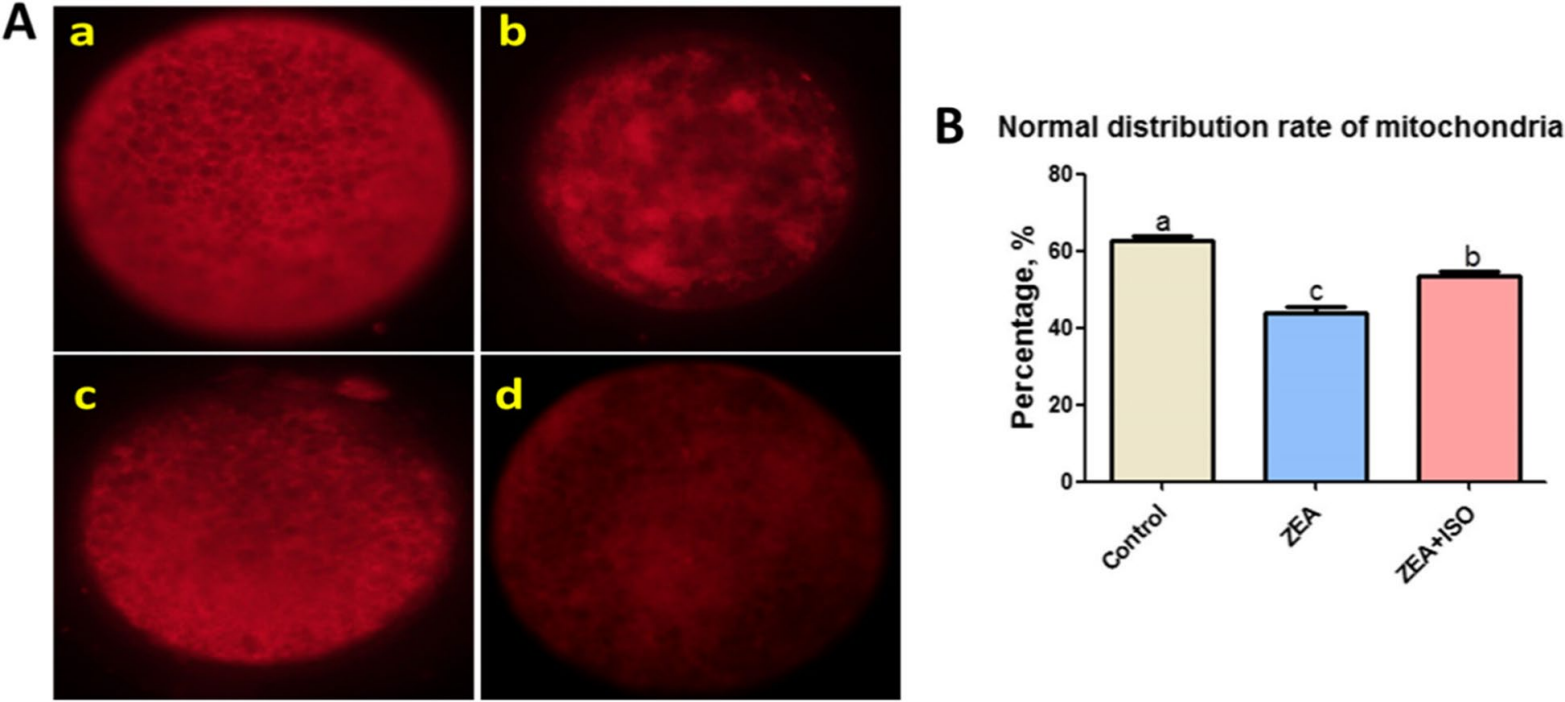
Isorhamnetin improved mitochondrial distribution in ZEA-exposed porcine oocytes. Oocytes were treated with ZEA (5 μmol/L) for 44 h in the presence or absence of isorhamnetin (10 μmol/L). **A** Representative photomicrographs of mitochondrial distribution. (a) Normal distribution. (b) Accumulative distribution. (c) Half edge distribution. (d) Intermediate distribution. **B** Percentage of normal distribution of mitochondria in Control, ZEA and ZEA + ISO. Various letters (*P* < 0.05) designate significant differences. Data are presented by average ± SEM

Mitochondria are important subcellular targets of ROS-induced cell damage. Nextly, the effect of isorhamnetin on mitochondrial dysfunction (including mitochondrial membrane potential and mitochondria distribution) in ZEA-exposed oocytes was analyzed by JC-1 and Mitotracker red staining. As shown in Figs. 4 and 5, compared with the control group, the red/green fluorescence ratio (*P* < 0.05, Fig. 4A–B) and mitochondrial normal distribution rate (*P* < 0.05, Fig. 5A–B) in ZEA group were significantly decreased. Meanwhile, compared with ZEA group, the red/green fluorescence ratio (Fig. 4A–B) and mitochondrial normal distribution rate (Fig. 5A–B) were significantly increased in ZEA + ISO group (*P* < 0.05). These results indicated the beneficial role of isorhamnetin in ZEA-exposed oocytes damage was positively correlated with the inhibition of mitochondrial dysfunction.

### Isorhamnetin suppressed ZEA-induced ER stress in porcine oocytes

**Fig. 6:**
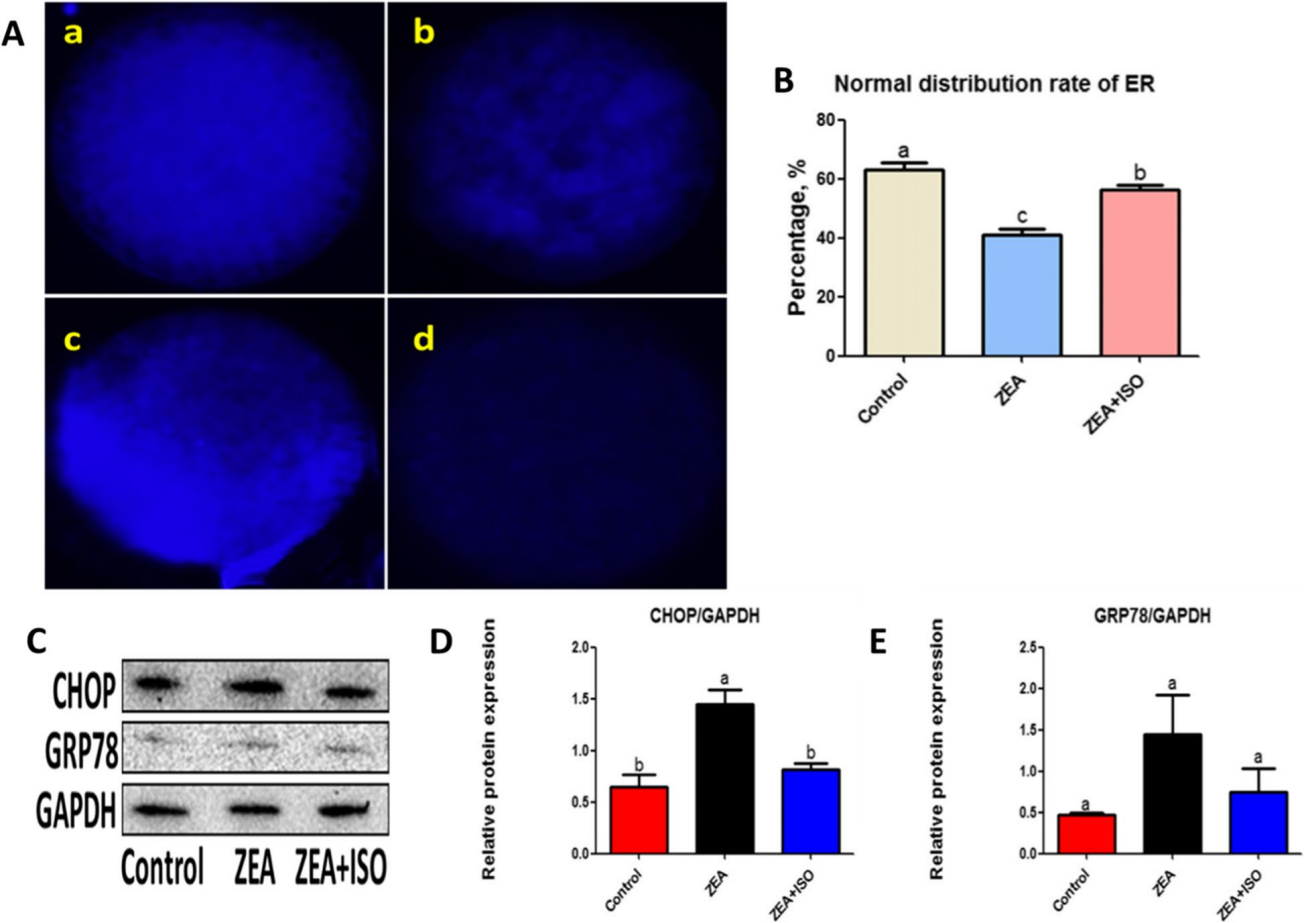
Isorhamnetin suppressed ZEA-induced ER stress in porcine oocytes. Oocytes were treated with ZEA (5 μmol/L) for 44 h in the presence or absence of isorhamnetin (10 μmol/L). **A** Representative images of ER distribution patterns. (a) Normal distribution. (b) Accumulative distribution. (c) Half edge distribution. (d) Almost no ER signal distribution. **B** Percentage of normal distribution of ER in Control, ZEA and ZEA + ISO. **C** Western blot of CHOP and GRP78 protein in Control, ZEA and ZEA + ISO. **D**–**E** Ratios of CHOP and GRP78 to GAPDH expression, respectively. Various letters (*P* < 0.05) designate significant differences. Data are presented by average ± SEM

Porcine oocytes were treated with ER-Tracker Blue to observe the effect of isorhamnetin on ER stress induced by ZEA. As shown in Fig. 6, compared with the control group (63.19% ± 2.15%) and ZEA + ISO group (56.1% ± 1.71%), the normal distribution rate of ER in ZEA-exposed oocytes (41.15% ± 1.94%, *P* < 0.01; Fig. 6A–B) was significantly decreased. Then, the expression of ER-associated marker proteins of CHOP and GRP78 were detected. We found that CHOP protein expression was significantly increased in ZEA-exposed oocytes, which was abolished by isorhamnetin (*P* < 0.05; Fig. 6C–D). However, there was no significant difference in GRP78 protein expression between ZEA group and ZEA + ISO group (*P* = 0.16; Fig. 6C and E). These results suggest that the isorhamnetin supplementation can effectively reverse ER-stress induced by ZEA exposure in porcine oocytes.

### Isorhamnetin relieved ZEA-caused damage through the PI3K/Akt signaling pathways

**Fig. 7:**
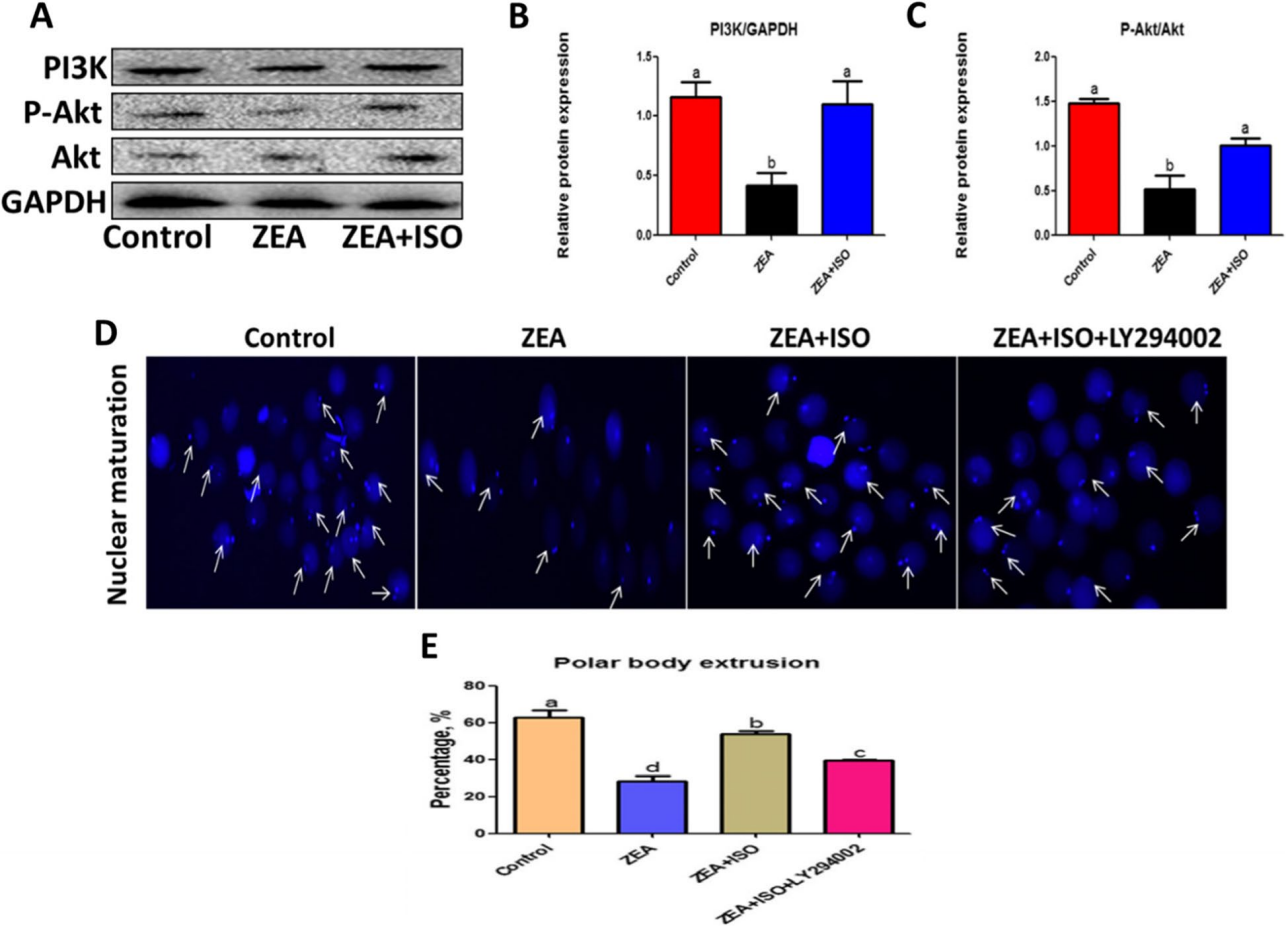
Isorhamnetin activated PI3K/Akt pathway in response to ZEA-induced meiosis arrest. Oocytes were co-cultured in ZEA (5 μmol/L), ISO (10 μmol/L) and LY294002 (20 μmol/L) for 44 h. **A** Western blot of PI3K, P-Akt and Akt protein in Control, ZEA and ZEA + ISO. **B**–**C** The ratios of PI3K to GAPDH and P-Akt to Akt expression were normalized, respectively. **D** Representative pictures of nuclear maturation in Control, ZEA, ZEA + ISO and ZEA + ISO + LY294002. **E** The percentage of polar extrusion was calculated. Various letters (*P* < 0.05) designate significant differences. Data are presented by average ± SEM

To clarify the protective mechanism of isorhamnetin in alleviating ZEA-caused oocyte damage. The activated forms of PI3K, Akt and p-Akt were detected in porcine oocytes. As shown in Fig. 7, PI3K and p-Akt protein expression were significantly decreased in ZEA group (*P* < 0.05; Fig. 7A–C). This action was alleviated by isorhamnetin treatment (*P* < 0.05; Fig. 7A–C).

**Fig. 8:**
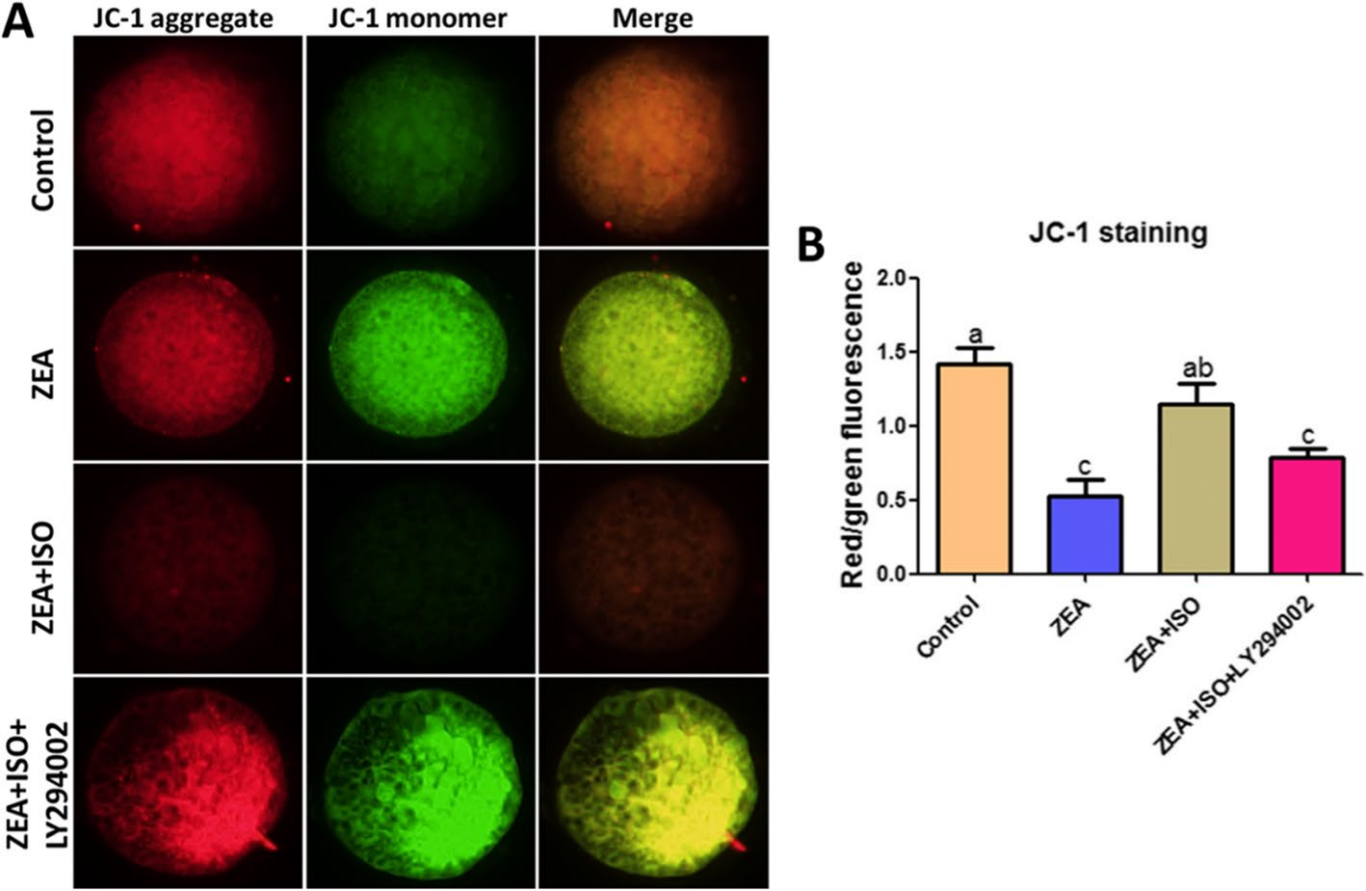
Isorhamnetin repaired ZEA-damaged mitochondrial membrane potential in a PI3K/Akt dependent manner. Oocytes were co-cultured in ZEA (5 μmol/L), ISO (10 μmol/L) and LY294002 (20 μmol/L) for 44 h. **A** Representative pictures of mitochondrial membrane potential in Control, ZEA, ZEA + ISO and ZEA + ISO + LY294002. **B** The fluorescence pixel ratio (red/green) was calculated. Various letters (*P* < 0.05) designate significant differences. Data are presented by average ± SEM

**Fig. 9:**
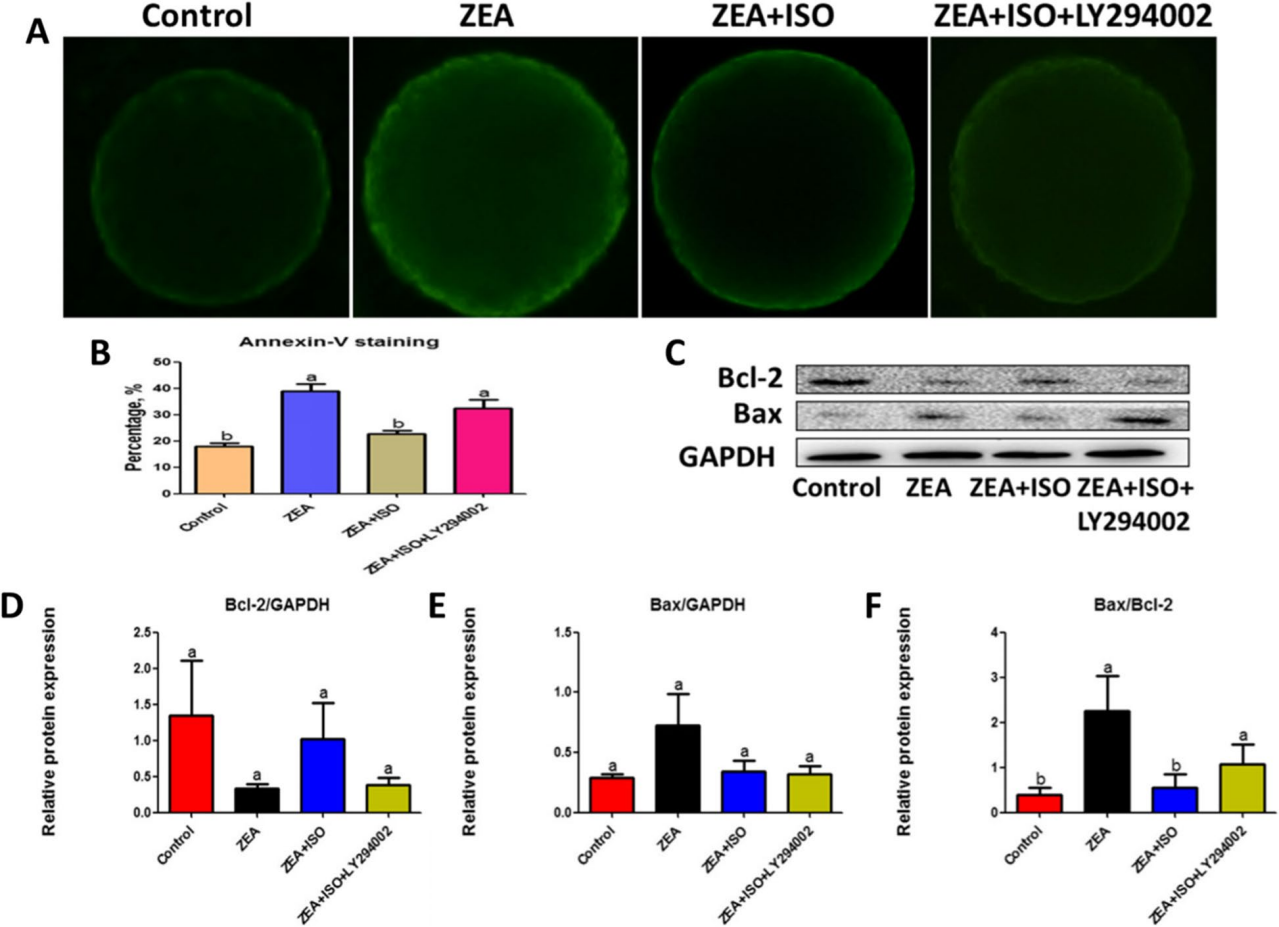
Isorhamnetin inhibited ZEA-caused apoptosis through the PI3K/Akt signaling pathway.Oocytes were co-cultured in ZEA (5 μmol/L), ISO (10 μmol/L) and LY294002 (20 μmol/L) for 44 h. **A** Representative pictures of apoptosis in Control, ZEA, ZEA + ISO and ZEA + ISO + LY294002. **B** The percentage of positive Annexin-V. **C** Western blot of Bcl-2 and Bax protein in Control, ZEA, ZEA + ISO and ZEA + ISO + LY294002. **D**–**F** The ratios of Bcl-2 to GAPDH, Bax to GAPDH and Bax to Bcl-2 expression were normalized, respectively. Various letters (*P* < 0.05) designate significant differences. Data are presented by average ± SEM

To determine whether isorhamnetin protected ZEA-induced damage was dependent on PI3K/Akt signaling pathway activity, the PI3K antagonist (LY294002) was cultured with ZEA and ISO in COCs for 44 h, and then the nuclear maturation and apoptosis of oocyte were evaluated. As shown in Fig. 7D and E, the beneficial effect of isorhamnetin on nuclear maturation was blocked by LY294002 (*P* < 0.05). In addition, isorhamnetin inhibited ZEA-induced oocyte apoptosis (*P* < 0.05; Fig. 9A–B) by increasing mitochondrial membrane potential (*P* < 0.05; Fig. 8A–B) and decreasing Bax/Bcl-2 protein expression (*P* < 0.05; Fig. 9C and F), which was eliminated by LY294002 (Figs. 7, 8 and 9). Together, these findings suggest that isorhamnetin protects oocytes from ZEA-induced damage by activating the PI3K/Akt signaling pathways.

## Discussion

ZEA widely exists in moldy grains and causes irreversible damage to the reproductive system of animals and humans [32]. Pigs are the most sensitive to ZEA toxicity in all animals [33]. Studies have shown that ZEA destroyed meiosis of porcine oocytes by interfering with the initial state of chromatin [14] and spindle malformation [13]. Up to now, there is no effective antidote for ZEA. Thus, it is necessary to explore potential compounds that can effectively protect ZEA-induced oocyte damage. Isorhamnetin (flavonoid compound) has a wide variety of pharmacological actions (anti-tumor, anti-oxidation, anti-bacterial, anti-inflammation and anti-virus) [34]. Isorhamnetin is also involved in cellular physiological processes such as proliferation and hormone secretion of porcine ovarian granulosa cells [28]. However, it is unclear whether isorhamnetin can protect porcine oocytes from ZEA-induced damage. Here, we provided direct evidence that isorhamnetin protected oocytes from ZEA-induced damage through promoting meiotic maturation and mitochondrial function, and inhibiting early apoptosis, oxidative stress and ER stress.

ZEA interferes with oocyte development, which is manifested as reduced oocyte maturation rate and abnormal oocyte morphology [32]. ZEA induced abnormal spindle and inhibited maturation of pig oocyte [16]. Lai et al. found that ZEA destroyed oocyte morphology and inhibited nuclear and cytoplasmic maturation of porcine oocytes [16]. ZEA also induced apoptosis of mouse Leydig cells by decreasing the expression of antiapoptotic protein Bcl-2 and increasing the expression of proapoptotic protein Bax [35]. In preliminary assays, we observed that ZEA inhibited the polar body extrusion of oocytes in a dose-dependent manner. Previous studies have shown that 5 μmol/L ZEA exposure induced meiotic arrest in porcine oocytes [15]. Similarly, we found that ZEA significantly inhibited the polar extrusion of oocytes in 3, 5, 8 and 10 μmol/L treatment groups. Based on previous studies [15] and our results, we chose 5 μmol/L ZEA as the model dose. Fortunately, isorhamnetin could effectively improve the decrease of porcine oocyte maturation induced by ZEA. Additionally, isorhamnetin inhibited the protein expression of Bax/Bcl-2 and C-Casp3 to prevent early apoptosis of porcine oocytes induced by ZEA. In recent years, natural flavonoids (antioxidant activity) have attracted extensive attention in promoting oocyte development. Quercetin significantly increased the proportion of porcine oocyte developing into blastocysts (24.3% vs. 16.8%) [36]. Yao et al. reported that kaempferol could alleviate the reduction of developmental competence of porcine oocytes during aging by improving mitochondrial function and decreasing oxidative stress [37]. The above data suggest that isorhamnetin alleviates the ZEA-induced damage by promoting nuclear maturation and inhibiting apoptosis of oocytes.

ZEA induces oxidative stress and stimulates ROS generation, and the excessive ROS production promotes ZEA-induced cell death [38]. Xu et al. reported that ZEA (10 μmol/L) caused oxidative stress in porcine embryos by increasing ROS levels [39]. Recent studies have shown that ZEA caused oxidative stress by inhibiting the expression of SOD2, GPX1, SOD1 and CAT proteins, thereby impairing placental function in rats [40]. Consistently, we observed that ZEA exposure increased ROS production, decreased SOD2 protein level, and triggered oxidative stress in oocytes. As expected, isorhamnetin repaired ZEA-induced oxidative stress in porcine oocytes. In fact, isorhamnetin itself is a potential antioxidant. Our previous studies have shown that isorhamnetin inhibited oxidative stress in porcine granulosa cells by decreasing ROS production and increasing SOD2 protein expression [28]. Similarly, isorhamnetin prevented oxidative stress in human retinal pigment epithelial cells by reducing the production of ROS [41]. These data show that isorhamnetin protects against ZEA-induced oxidative stress by increasing SOD2 protein expression and decreasing ROS production.

Cumulus cells control meiotic resumption of oocytes by regulating follicular microenvironment [42]. In addition, the expansion degree of COCs can be used as the morphological standard of porcine oocyte maturation [42]. Pang et al. reported that paraquat significantly inhibited COCs expansion in bovine oocytes by decreasing the expression of cumulus expansion-related genes (*PTGS2, TNFAIP6, PTX3* and *HAS2*) [43]. In vitro culture of porcine oocytes, ZEA (5 μmol/L) treatment group (0.042 ± 0.024 mm^2^) significantly reduced the expansion degree of COCs compared with the control group (0.117 ± 0.036 mm^2^) [15]. Consistently, our data showed that ZEA significantly inhibited the expansion of cumulus cells around porcine oocytes.

It has been reported that the expansion of cumulus cells was closely related to organelles rearrangement of porcine oocytes [44]. First of all, the function of mitochondria in porcine oocytes was studied. Mitochondrial dysfunction can destroy the female reproductive function of oocyte maturation, fertilization and embryonic development [45]. Strong evidence indicated that ZEA damaged Sertoli cells through reducing mitochondrial membrane potential and altering mitochondrial structure [46]. ZEA also leads to necrosis and apoptosis of porcine granulosa cells by damaging mitochondrial transmembrane potential [47]. Wang et al. reported that ZEA decreased mitochondrial membrane potential, normal distribution and the expression of mitochondrial-related genes (*PGC1a, ATP5B, pdss2* and *pdss1*) to inhibit meiosis of porcine oocytes [15]. Consistently, we found that ZEA inhibited the red/green fluorescence ratio and normal distribution rate of mitochondria. As expected, isorhamnetin recovered ZEA-induced mitochondrial dysfunction in oocytes. Indeed, isorhamnetin protected hepatocytes from arachidonic acid (AA) + iron by inhibiting mitochondrial dysfunction [48]. Meanwhile, isorhamnetin promoted the expression of *PGC-1* gene and prevented obesity-related mitochondrial dysfunction in 3T3-L1 cells [49]. These findings suggest that isorhamnetin can protect porcine oocytes from ZEA induced mitochondrial dysfunction.

The correct folding of functional proteins in ER is a prerequisite for maintaining oocyte maturation [50]. GRP78 is considered as a marker protein of ER stress and plays a key role in protein folding [51]. ER stress plays a key role in ZEA-induced reproductive cytotoxicity [52]. Previous results showed that ZEA disrupted ER distribution and decreased *ATF4* and *CHOP* gene expression to induce ER stress in porcine oocytes [15]. In addition, ZEA induced Leydig cells death in goat and mouse through ER stress [53, 54]. In this study, ZEA induces ER stress in porcine oocytes by activating CHOP and GRP78 protein expression. However, isorhamnetin played a protective role in response to ZEA-induced ER stress. Similarly, Zheng et al. reported that isorhamnetin decreased CHOP and GRP78 protein expression and inhibited pulmonary ER stress in mice [55]. Isorhamnetin also suppressed ER stress-induced apoptosis in N2a cells [56]. These results indicate that isorhamnetin alleviates ZEA-induced ER stress by increasing normal ER distribution and inhibiting ER stress-related protein expression.

PI3K/Akt plays an important role in oocyte maturation, granulosa proliferation and primordial follicle recruitment [29]. Growing evidences showed that ZEA inhibited PI3K/Akt signaling pathway and destroyed the reproductive function of animals [57, 58]. Published data have suggested that ZEA promoted apoptosis of mouse Leydig cells by inhibiting PI3K/Akt signaling pathway [59]. Whole-transcriptome sequencing showed that the toxicity of ZEA to porcine ovarian granulosa cells was closely related to PI3K/Akt signaling pathway [31]. In this study, ZEA induced oocyte damage and inhibited PI3K/Akt signaling pathway. It was worth noting that isorhamnetin could regulate the nuclear maturation, mitochondrial membrane potential and apoptosis of porcine oocytes to repair the damage caused by ZEA. Interestingly, the beneficial effect of isorhamnetin on ZEA-caused damage was eliminated by PI3K inhibitors. The above data hinted that isorhamnetin recovered ZEA-caused porcine oocyte damage through the PI3K/Akt signaling pathway. Similar to this study, our previous studies showed that isorhamnetin increased the proliferation of porcine ovarian granulosa cells in a PI3K/Akt dependent manner [28]. Indeed, previous reports have also shown that PI3K/Akt signaling pathway played an important role in the regulation of inflammation [60], oxidative stress [41] and apoptosis [61] by isorhamnetin. In brief, these results suggest that isorhamnetin and PI3K/Akt signaling pathway have a functional interaction in porcine oocyte damage induced by ZEA.

## Conclusion

In conclusion, our study demonstrated that ZEA exposure induced reproductive toxicity during porcine oocyte maturation. Isorhamnetin prevented the ZEA-induced meiotic arrest, apoptosis, oxidative stress, mitochondrial dysfunction, ER stress through the PI3K/Akt signaling pathways, thereby alleviating oocytes damage caused by ZEA. These findings suggest that isorhamnetin has a great application prospect in alleviating ZEA-induced oocyte damage. Thus, animals and humans will be healthier, and feed products and foods will be safer.

## Supplementary Information

**Additional file 1.** Explanation for the low maturation rate of porcine oocytes.

**Additional file 2.** The raw western blot images.

## Data Availability

All data of the research results are within this paper.

## Abbreviations

COCs: Cumulus oocyte complexes
DMSO: Dimethyl sulfoxide
ER: Endoplasmic reticulum
FBS: Fetal bovine serum
IVM: In vitro maturation
PBS: Phosphate-buffered saline
ROS: Reactive oxygen species
ZEA: Zearalenone
