# Supplementary material for: Isorhamnetin protects porcine oocytes from zearalenone-induced reproductive toxicity through the PI3K/Akt signaling pathway
Source: J Anim Sci Biotechnol. 2023 Feb 3;14:22. doi: 10.1186/s40104-022-00809-w (PMC9896747; doi:10.1186/s40104-022-00809-w)
Supplement: Supplementary file 1 — Additional file 1. Explanation for the low maturation rate of porcine oocytes. [file 40104_2022_809_MOESM1_ESM.docx]

**Explanation for the low maturation rate of porcine oocytes in our manuscript:** the quality of our oocyte samples was not as good as before due to the change in the source of porcine in the abattoir where we sampled them. In previous articles published in our laboratory, the maturation rate of oocytes was all about 60%. For example, Duan et al. [1, 2] and Chen et al. [3]. In addition, the maturation rate of porcine oocytes was also about 60% in the published studies of other scholars [4, 5]. It was worth noting that the maturation rate of porcine oocytes had also reached 75%–80% in the repeated process of our experiment, but the repeatability was not very stable (It was highly correlated with the quality of the oocytes that we collected each time). The maturation rate of porcine oocytes in our manuscript was a reproducible and reliable data. Although the maturity rate was not very high, the experimental results were absolutely true.

**References**

1. Duan J, Chen H, Li Y, Xu D, Li X, Zhang Z, et al. 17β-estradiol enhances porcine meiosis resumption from autophagy-induced gap junction intercellular communications and connexin 43 phosphorylation via the MEK/ERK signaling pathway. J Agric Food Chem. 2021;69(40): 11847–55. https://doi.org/10.1021/acs.jafc.1c04212.

2. Duan J, Chen H, Xu D, Li Y, Li X, Cheng J, et al. 17β-estradiol improves the developmental ability, inhibits reactive oxygen species levels and apoptosis of porcine oocytes by regulating autophagy events. J Steroid Biochem Mol Biol. 2021;209:105826. https://doi.org/10.1016/j.jsbmb.2021.105826.

3. Chen H, Cheng J, Yang Y, Li Y, Jiang X, Yang L, et al. Phospholipase C inhibits apoptosis of porcine oocytes cultured in vitro. J Cell Biochem. 2020;121(7):3547-3559. https://doi.org/10.1002/jcb.29636.

4. Yin C, Liu J, Chang Z, He B, Yang Y, Zhao R. Heat exposure impairs porcine oocyte quality with suppressed actin expression in cumulus cells and disrupted F-actinformation in transzonal projections. J Anim Sci Biotechnol. 2020;11:71. https://doi.org/10.1186/s40104-020-00477-8.

5. Yang X, Miao Y, Cui Z, Lu Y, Zhou C, Zhang Y, et al. Casein kinase 2 modulates the spindle assembly checkpoint to orchestrate porcine oocyte meiotic progression. J Anim Sci Biotechnol. 2020;11:31. https://doi.org/10.1186/s40104-020-00438-1.
