# Supplementary material for: Isorhamnetin protects porcine oocytes from zearalenone-induced reproductive toxicity through the PI3K/Akt signaling pathway
Source: J Anim Sci Biotechnol. 2023 Feb 3;14:22. doi: 10.1186/s40104-022-00809-w (PMC9896747; doi:10.1186/s40104-022-00809-w)

**The raw western blot images are shown below:**

**Fig. 2** Isorhamnetin inhibited ZEA-caused apoptosis

1. Protein is Bcl-2. The different treatments from left to right are Control, ZEA, ZEA+5 μmol/L ISO, ZEA+10 μmol/L ISO and ZEA+20 μmol/L ISO, respectively.


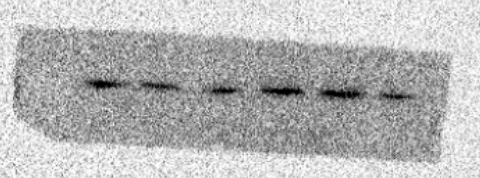


1. Protein is Bax. The different treatments from left to right are Control, ZEA, ZEA+5 μmol/L ISO, ZEA+10 μmol/L ISO and ZEA+20 μmol/L ISO, respectively.


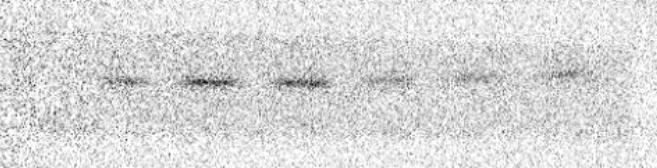


1. Protein is C-Casp3. The different treatments from left to right are Control, ZEA, ZEA+5 μmol/L ISO, ZEA+10 μmol/L ISO and ZEA+20 μmol/L ISO, respectively.


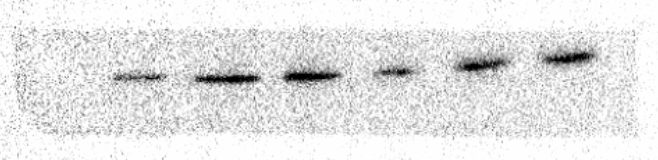


1. Protein is GAPDH. The different treatments from left to right are Control, ZEA, ZEA+5 μmol/L ISO, ZEA+10 μmol/L ISO and ZEA+20 μmol/L ISO, respectively.


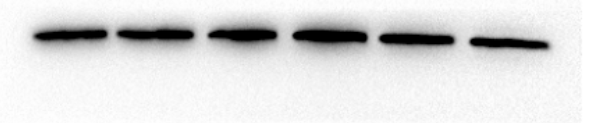


**Fig. 3** Isorhamnetin relieved oxidative stress caused by ZEA

1. Protein is SOD2. The different treatments from left to right are Control, ZEA and ZEA+ISO, respectively.


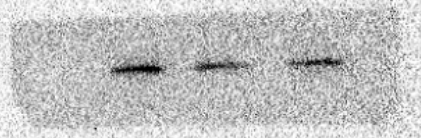


2. Protein is GAPDH. The different treatments from left to right are Control, ZEA and ZEA+ISO, respectively.


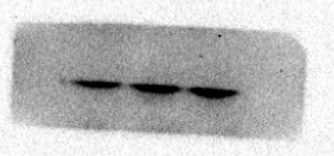


**Fig. 6** Isorhamnetin suppressed ZEA-induced ER stress in porcine oocytes

1. Protein is CHOP. The different treatments from left to right are Control, ZEA and ZEA+ISO, respectively.


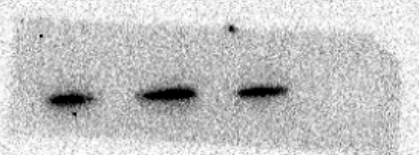


2. Protein is GRP78. The different treatments from left to right are Control, ZEA and ZEA+ISO, respectively.


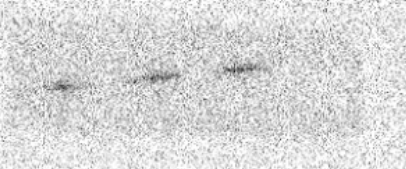


3. Protein is GAPDH. The different treatments from left to right are Control, ZEA and ZEA+ISO, respectively.


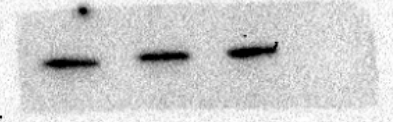


**Fig. 7** Isorhamnetin activated PI3K/Akt pathway in response to ZEA-induced meiosis arrest

1. Protein is PI3K. The different treatments from left to right are Control, ZEA and ZEA+ISO, respectively.


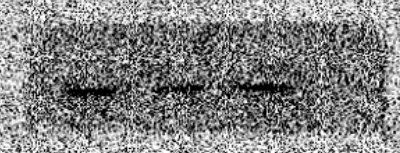


2. Protein is P-Akt. The different treatments from left to right are Control, ZEA and ZEA+ISO, respectively.


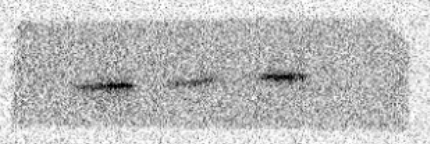


3. Protein is Akt. The different treatments from left to right are Control, ZEA and ZEA+ISO, respectively.


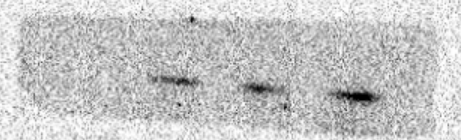


4. Protein is GAPDH. The different treatments from left to right are Control, ZEA, ZEA+ISO and ZEA+ISO+LY294002, respectively.


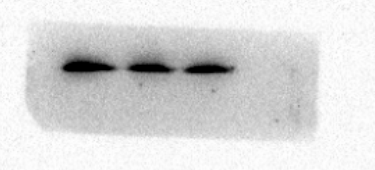


**Fig. 9** Isorhamnetin inhibited ZEA-caused apoptosis through the PI3K/Akt signaling pathway

1. Protein is Bcl-2. The different treatments from left to right are Control, ZEA, ZEA+ISO and ZEA+ISO+LY294002, respectively.


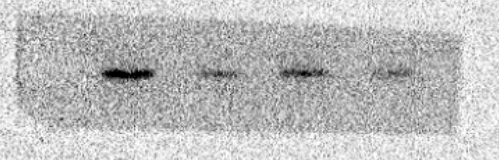


2. Protein is Bax. The different treatments from left to right are Control, ZEA, ZEA+ISO and ZEA+ISO+LY294002, respectively.


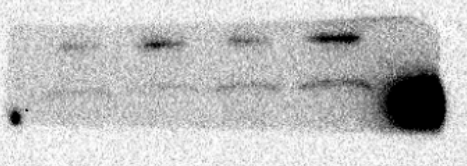


3. Protein is GAPDH. The different treatments from left to right are Control, ZEA, ZEA+ISO and ZEA+ISO+LY294002, respectively.


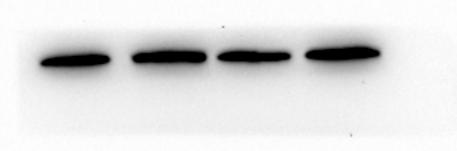

Supplement: Supplementary file 2 — Additional file 2. The raw western blot images. [file 40104_2022_809_MOESM2_ESM.docx]
